# The Microbiome and *Coxiella* Diversity Found in *Amblyomma hebraeum* and *Dermacentor rhinocerinus* Ticks Sampled from White Rhinoceros

**DOI:** 10.1007/s00248-025-02549-6

**Published:** 2025-05-22

**Authors:** Jemma K. Mitchell, Sonja Matthee, Andrew Ndhlovu, Michele Miller, Peter Buss, Conrad A. Matthee

**Affiliations:** 1 Evolutionary Genomics Group, Department of Botany and Zoology, Stellenbosch University, Stellenbosch, South Africa; 2 Department of Conservation Ecology & Entomology, Stellenbosch University, Stellenbosch, South Africa; 3 School for Climate Studies, Stellenbosch University, Private Bag X1, Matieland, 7602 South Africa; 4 South African Medical Research Council Centre for Tuberculosis Research, Division of Molecular Biology and Human Genetics, Faculty of Medicine and Health Sciences, Stellenbosch University, PO Box 241, Cape Town, 8000 South Africa; 5 Veterinary Wildlife Services, South African National Parks, Kruger National Park, Private Bag X402, Skukuza, 1350 South Africa

**Keywords:** *Coxiella burnetii*, *Ceratotherium simum*, Kruger National Park, *IS1111*, Tick microbiome, 16S rRNA

## Abstract

The microbiome and the prevalence of the pathogenic bacterium *Coxiella burnetii* in ticks associated with white rhinoceros, *Ceratotherium simum*, is unknown. Targeted Illumina 16S rRNA amplicon sequencing was used to characterize the bacterial microbiome diversity found within 40 *Amblyomma hebraeum* and 40 *Dermacentor rhinocerinus* ticks collected from 40 white rhinoceros individuals in the Kruger National Park, South Africa. Specific emphasis was also given to further investigate the prevalence of the pathogenic *C. burnetti* in these tick species. At the phylum level, Proteobacteria dominated both tick microbiomes, followed by Actinobacteria and Firmicutes; *Coxiella* was the most abundant genus within *A. hebraeum* and *Rickettsia* within *D. rhinocerinus*. While alpha diversity did not differ significantly between the two tick species, beta diversity revealed significant species-specific differences in bacterial community composition. Additionally, there was no correlation between sampling region and microbiome diversity or composition for either tick species. Twenty-five *Coxiella* amplicon sequence variants (ASVs) were identified, forming three distinct monophyletic *Coxiella* clades and a fourth single ASV lineage. The *Coxiella* clades showed a correlation to tick species identity with *D. rhinocerinus* harboring significantly greater *Coxiella* diversity than *A. hebraeum—*potentially indicative of different coevolutionary pathways between the bacteria and their respective hosts. PCR of the *IS1111* transposase gene for 238 ticks detected a 66.1% (56.7–74.4%) prevalence for *C. burnetii* in *D. rhinocerinus* compared to 55.8% in *A. hebraeum* (46.5–64.8%). These findings support a notion that each tick species is characterized by its own microbiome community composition and that both *A. hebraeum* and *D. rhinocerinus* may act as reservoirs and potential vectors of *C. burnetii* to white rhinoceros.

**Supplementary Information:**

The online version contains supplementary material available at 10.1007/s00248-025-02549-6.

## Introduction

Ticks are important vectors of disease capable of transmitting a variety of microbes to humans, livestock, and wildlife [1, 2]. These microbes play an integral role in tick fitness, survival, and vectoral capacity and include a diverse array of endosymbiotic, commensal, and pathogenic bacteria [1, 3, 4]. Genera such as *Anaplasma*, *Borrelia*, *Coxiella*, *Ehrlichia*, and *Rickettsia* are known to have strains that are pathogenic [2, 5]. The gram-negative intracellular bacterium, *Coxiella burnetii*, which forms the focus of the present study, is well known to cause coxiellosis or Q fever and can affect humans, wildlife, and livestock [6–8]. Many infected individuals remain asymptomatic, but since the main sites of chronic infection are the mammary glands and uterus [9], the disease is often associated with reproductive challenges including mastitis, metritis, weak offspring, premature delivery, infertility, stillbirth, and abortion [6].

In addition to the pathogenic *C. burnetti*, there are also highly prevalent *Coxiella* endosymbionts which have a broad tick host range [10, 11]. This lack of knowledge is particularly true for ticks occurring on wildlife [10, 12]. Interestingly, phylogenetic analysis of *Coxiella* sequences found within ticks revealed that the pathogenic *C. burnetii* likely evolved from a maternally inherited *Coxiella* endosymbiont [13]*.* As obligate blood-feeders, ticks rely on these endosymbiotic microbes to supplement nutrients and vitamins lacking in their hematophagous diet, and apart from *Coxiella*, other endosymbionts include *Rickettsia*, *Francisella*, and *Candidatus midichloria* [14].

White rhinoceros (*Ceratotherium simum*) are an iconic flagship species currently listed by the IUCN Red List of Threatened Species (IUCN, 2022) as near threatened [15]. Rhinoceros are susceptible to infection by *C. burnetii*, but the exact route of infection is poorly understood [16]. Coxiellosis was recently identified and associated with abortions in a managed group of white rhinoceros in the USA [17]. In South Africa, Donnelly et al. [16] reported an overall seropositivity of 71.0% for *C. burnetii* in white rhinoceros sampled in Kruger National Park (KNP; historically home to the largest white rhinoceros population globally) while individuals sampled in surrounding private reserves had a lower seropositivity of 41.1%.

Since the KNP has served as a source population for regional rhinoceros re-introductions into Africa, it is important to investigate the microbial diversity and abundance and specifically the incidence of *C. burnetii* in tick species associated with rhinoceros [16]. In South Africa, *C. burnetii* has been detected in several ixodid ticks associated with domestic animals, and these include *Rhipicephalus evertsi evertsi*, *Rhipicephalus sanguineus*, *Haemaphysalis elliptica*, and* Amblyomma hebraeum* [18–20]. To further our knowledge on the microbiome of ticks associated with wildlife and in particular to gain more insights into the prevalence and diversity of *Coxiella*, we sampled two ixodid tick species (*Dermacentor rhinocerinus* and *A. hebraeum*) commonly associated with white rhinoceros [21]. These ticks were the most abundant during sampling and the life history of the two species also differ. *Amblyomma hebraeum* is a generalist species (adult stages can occur on multiple different mammalian host species) while adult stages of *D. rhinocerinus* occur only on rhinoceros [21]. *Amblyomma hebraeum* is a well-known vector of disease and can harbor an array of pathogenic bacteria such as *C. burnetii*, *Rickettsia africae*, and *Ehrlichia ruminantium* [21–24]. While there is nothing known about the microbiome diversity or vectoral capacity of *D. rhinocerinus*, a study on the congeneric *D. reticulatus* in Europe detected *Anaplasma marginale*, *C. burnetii* as well as several species of *Rickettsia*, *Borrelia*, *Francisella*, and *Bartonella* [25].

To obtain novel data describing the microbiome composition found within *A. hebraeum* and *D. rhinocerinus*, we sampled ticks from white rhinoceros in KNP and embarked on a 16S rRNA metagenetic study. To further investigate the prevalence of the pathogenic *C. burnetti* in ticks associated with white rhinoceros in KNP, we used species-specific amplification of the *IS1111* transposase gene. Since different tick species often differ in microbiome composition, it was hypothesized that the generalist *A. hebraeum* and the specialist *D. rhinocerinus* would differ significantly in microbiome species compositions [26–28]. In addition, since the environment off the host can play a role in shaping microbiome diversity and abundance [28–32], it was also predicted that ticks sampled from different sampling regions (landscape types) within KNP would have different microbiome diversities and abundances. Finally, it was hypothesized that *Coxiella* bacteria (symbionts and pathogenic strains) would be highly prevalent in both tick species [3, 11, 33] and since members of the *Dermacentor* genus have been found to be one of the most common carriers of the bacterium, *C. burnetii* would be more prevalent in *D. rhinocerinus* [34].

## Materials and Methods

### Tick Sampling

Ticks were opportunistically sampled from white rhinoceros during dehorning procedures and/or health assessments in the KNP during 2019–2023. Sampled ticks were dry-frozen at –80 °C. Tick identification, sorting, and subsequent DNA extraction took place in a sterile environment at the Veterinary Wildlife Services Laboratory, Skukuza, KNP. Ticks were identified to species level based on morphology [21] using a Nikon stereo microscope (Nikon Microscopy, Tokyo, Japan). For microbiome analysis, 40 *A. hebraeum* adult males and 40 *D. rhinocerinus* adult males were respectively sampled from 40 different white rhinoceros individuals (one tick from each species were sampled from the same rhinoceros individual). Ten of the rhinoceros individuals originated from the central region and 30 individuals from the southern region (Supplementary Fig. 1). To determine *C. burnetii* prevalence, an additional 158 tick samples were included (80 *A. hebraeum* and 78 *D. rhinocerinus* sampled randomly from different rhinoceros individuals). 

### DNA Extraction and Microbiome Sequencing

To remove external surface contamination, ticks were washed five times prior to DNA extraction. First, a 10% sodium hypochlorite (NaClO) rinse was followed by a sterile phosphate buffered saline (PBS) wash and then an absolute ethanol (100% EtOH) wash. The ticks were then double-rinsed with 1 mL of sterile PBS to remove remaining ethanol. Each wash was conducted for 1 min using 1 mL of solution and a vortex. Ticks were cut into pieces in a sterile petri dish and DNA extractions were performed in separate sterile 1.5-mL Eppendorf tubes. To prevent contamination, tick cutting and DNA extractions were performed in a laminar flow hood using separate new sterile scalpels and filter tips.

Genomic DNA was extracted using the NucleoSpin Tissue kit (Macherey–Nagel, Düren, Germany). First, 180 μL of the NucleoSpin Tissue lysis buffer (Macherey–Nagel) and 25 μL of Proteinase K (Macherey–Nagel) were added to the samples and incubated overnight at 56 °C. A negative DNA extraction (blank) without any tick tissue was also included. DNA was eluted in 100 μL elution buffer (Macherey–Nagel) and stored at − 20 °C.

Prior to sequencing and PCR screening, DNA concentration was determined using the Qubit dsDNA Broad Range Assay kit (Thermofisher Scientific, Waltham, MA, USA) and the Qubit 4 fluorometer (Thermofisher Scientific) as per the manufacturer’s instructions. DNA samples to be used for microbiome analysis were equilibrated to represent similar DNA concentration in all samples and these were analyzed by BGI Genomics, Hong Kong. The 16S rRNA V3–V4 hypervariable region was amplified using the 341 F (5′-ACT CCT ACG GGA GGC AG CAG-3′) and 806R (5′-GGA CTA CHV GGG TWT CTA AT-3′) fusion primers [35] and sequenced on an Illumina HiSeq platform (Illumina, San Diego, CA, USA).

### Species-Specific Conventional PCR Detection of *Coxiella burnetii*

Ticks were washed twice prior to DNA extraction, first using absolute ethanol (100% EtOH), followed by sterile PBS. The primers Trans1 (5′-TAT GTA TCC ACC GTA GCC AGT C-3′) and Trans2 (5′-CCC AAC AAC ACC TCC TTA TTC-3′), which target a 687-bp fragment of the *IS1111* transposase element of the *C. burnetii* genome [36], were used to screen the 238 tick samples (80 that were included in the microbiome study and 158 additional samples) for *C. burnetii.* We followed the protocol of Kamau et al. [36] with some modifications. Each 12.5-μL PCR reaction consisted of 1 μL equilibrated template DNA (the same concentration), 0.5 μL of each primer (final concentration of 0.5 μM), 6.25 μL of Taq DNA Polymerase Master Mix RED (Amplicon, Denmark), and 4.25 μL of nuclease free water. Amplification was performed on a GeneAmp PCR system 2700 thermal cycler (Applied Biosystems, USA). A total of 5 μL of each PCR product (including the positive and negative controls) was visualized on a 1% agarose gel stained with ethidium bromide. Eight randomly selected positive samples were Sanger sequenced in both directions at the Central Analytical Facility of Stellenbosch University to confirm the authenticity of the pathogenic *C. burnetii*. Sequences were manually edited using the BioEdit Sequence Alignment Editor (version 7.2.5) and then queried against known *C. burnetii* reference sequences in GenBank using the Basic Local Alignment Search Tool (BLASTn) online server. The PCR products of samples which produced any visible band at the expected amplicon size of 687 bp (also matching the fragment size of a sequenced positive control) were noted as being positive for *C. burnetii*. To test for PCR consistency and amplification accuracy, 30 samples (tested as either positive or negative) were randomly selected for reamplification, following the same procedure.

### Sequence Data Analyses

Sequenced reads were demultiplexed and reads with a Phred quality score of less than 20 over a 25-bp sliding window were truncated, together with reads whose lengths were less than 75% of their original lengths after truncation. Sequence quality was assessed using FastQC [37] and MultiQC [38]. Thereafter, the Quantitative Insights Into Microbial Ecology (QIIME2 2023.7) bioinformatics pipeline [39] was used to perform sequence analysis on the Stellenbosch University high performance computing cluster (HPC) 2 (http://www.sun.ac.za/hpc). The DADA2 plugin with default parameters was used for denoising, paired-end merging, removal of chimeric reads, sequence dereplication, and generation of amplicon sequence variants (ASVs). The Naive Bayes classifier trained on the Silva 138 database (99% OTUs full-length sequences) was used to assign genus level taxonomy to representative ASV sequences at 99% nucleotide sequence similarity. The ASVs were then aligned using the align-to-tree-mafft-fasttree pipeline and a phylogenetic tree was created using q2-phylogeny. Finally, the filter-features method from the q2-feature-table plugin was used to remove any ASVs with a total count of less than 10 over the whole dataset.

All further downstream processing was conducted in R (version 4.3.3 (2024–02–29)). The ASV table was filtered to exclude “Archaea,” “Eukaryota,” or “Chloroplast” using phyloseq [40]. Decontam v1.22.0 (Davis et al*.*, 2018) was used to identify and remove all contaminant sequences from the dataset under a prevalence method using a threshold of 0.1. To ensure an even sampling depth, the samples were rarefied to 48,000 sequences using the phyloseq::rarefy_even_depth function after inspecting rarefaction curves (Supplementary Fig. 2). All further statistical analyses were conducted on the rarefied ASV table.

### Statistical Analyses

Statistical analyses were performed using various R (version 4.3.3 (2024–02–29)) packages. Diversity analyses were performed at the ASV level using phyloseq v1.46.0 [41]. Alpha diversity was assessed for each tick species using (1) observed richness (not accounting for abundance or evenness), (2) Shannon diversity index (accounts for richness and evenness), and (3) Simpson diversity index (accounts for richness and evenness). Statistical significance for these were performed using the Wilcoxon rank-sum test. The relative abundance of the 10 most prevalent bacterial phyla as well as the 10 most prevalent bacterial genera were calculated using ASV counts. Alpha diversity measures and the relative abundances were displayed graphically using ggplot2 v3.5.1 [41]. Beta diversity, the Bray–Curtis dissimilarity index, and a principal coordinate analysis (PCoA) plot was used to visualize the bacterial community differences between the tick species. Significance for the latter was determined by using a permutational multivariate analysis of variance (PERMANOVA) conducted on the Bray–Curtis dissimilarity distances using the adonis2 function in vegan v2.6–6.1 [42]. Differentially abundant ASVs were calculated at the genus level using the Microbiome differential abundance and correlation analysis with bias correction (ANCOMBC v2.4.0) package [43]. Only ASVs present in at least 10% of the samples were included with the Holm’s method selected for *p*-adjustment. The tick samples were respectively grouped according to sampling region (central and southern regions of the KNP; Supplementary Fig. 1) and similar diversity analyses and statistical tests were performed as outlined above.

### *Coxiella* Phylogenic Analyses

Phylogenetic analysis of the *Coxiella* ASVs was performed using maximum likelihood (ML) and Bayesian inference (BI) in MEGA version 11 [44] and MrBayes 3.2.7a [45], respectively. Additional 16S rRNA reference sequences for *C. burnetii* and *Coxiella*-like organisms (CLO) were downloaded from GenBank (Supplementary Table 1). 16S rRNA sequences of *Rickettsiella* (two sequences) and *Legionella* were included as outgroups (Supplementary Table 1). Sequences were aligned using MUSCLE in MEGA and the same software was also used to determine the best-fitting evolutionary model based on the Akaike Information Criterion. For the ML, heuristic search nodal support was estimated using 100 bootstrap replicates, and posterior probabilities for the Bayesian analyses were obtained after running 5 million generations in parallel, sampling every 50 generations with a burn-in of 5000. Consensus trees were visualized using FigTree v1.4.4 (http://tree.bio.ed.ac.uk/software/figtree/).

### *Coxiella burnetii* Summary and Descriptive Statistics

Phyloseq was used to perform diversity analyses of *Coxiella* ASVs. Alpha and beta diversity calculations followed the same methodology outlined above. The number of ticks which tested positive for *C. burnetii* was summarized for each species, and the prevalence was expressed as a proportion by dividing the total number of positive samples by the total number of samples screened for each tick species. The prop.test function in R was used to calculate 95% confidence intervals for each species, and a chi-square test was performed to assess statistical significance. Prevalence was also summarized by region (central and southern) for each tick species, using the methods described above, with a Fisher’s exact test performed to test for significant differences.

## Results

### Microbiome Diversity and Relative Abundance within and among Tick Species

**Fig. 1:**
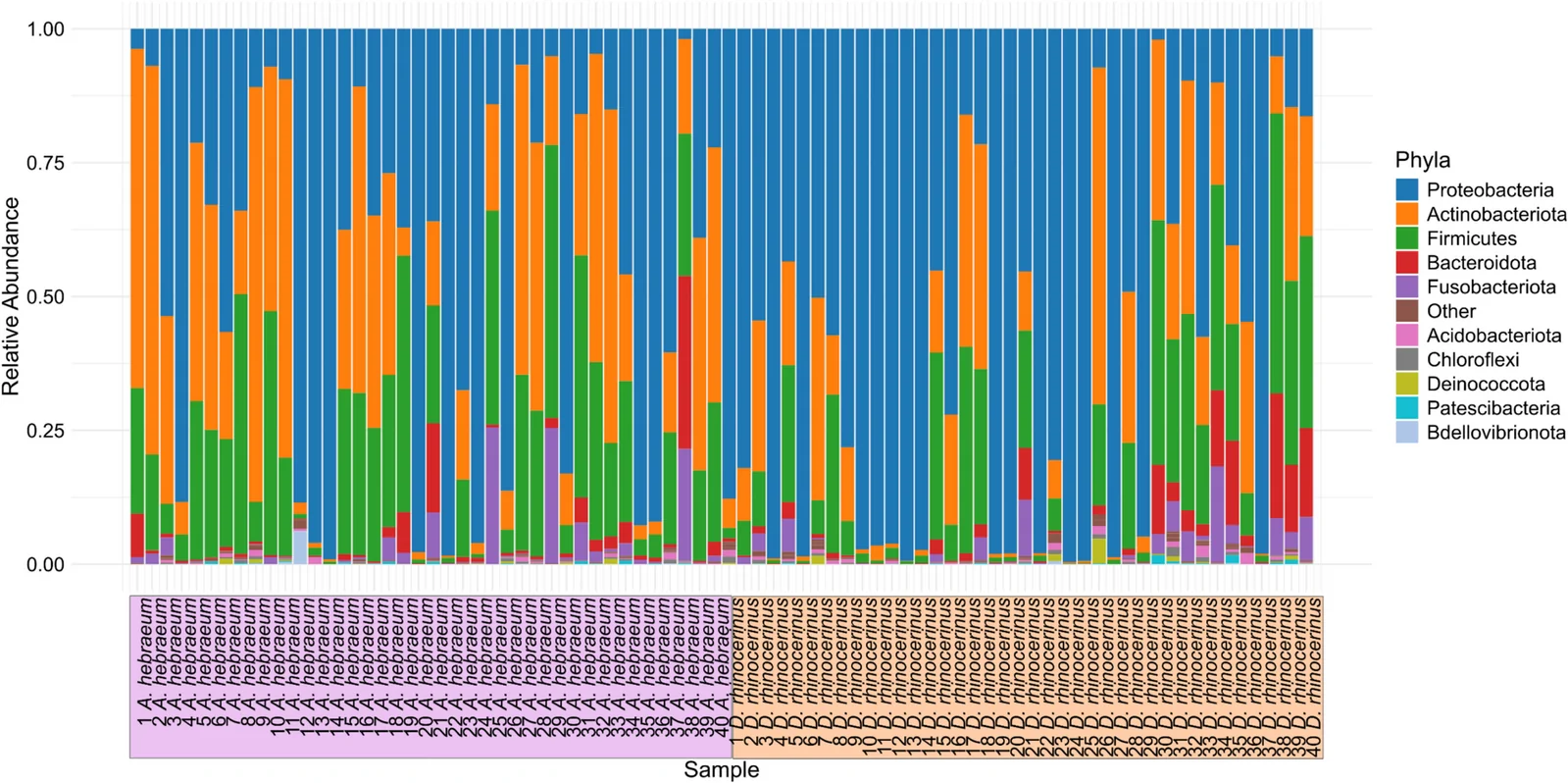
Relative abundance of the 10 most abundant phyla found across the 80 tick samples sequenced in the study. *Amblyomma hebraeum* samples are highlighted in purple and *Dermacentor rhinocerinus* samples in orange

**Fig. 2:**
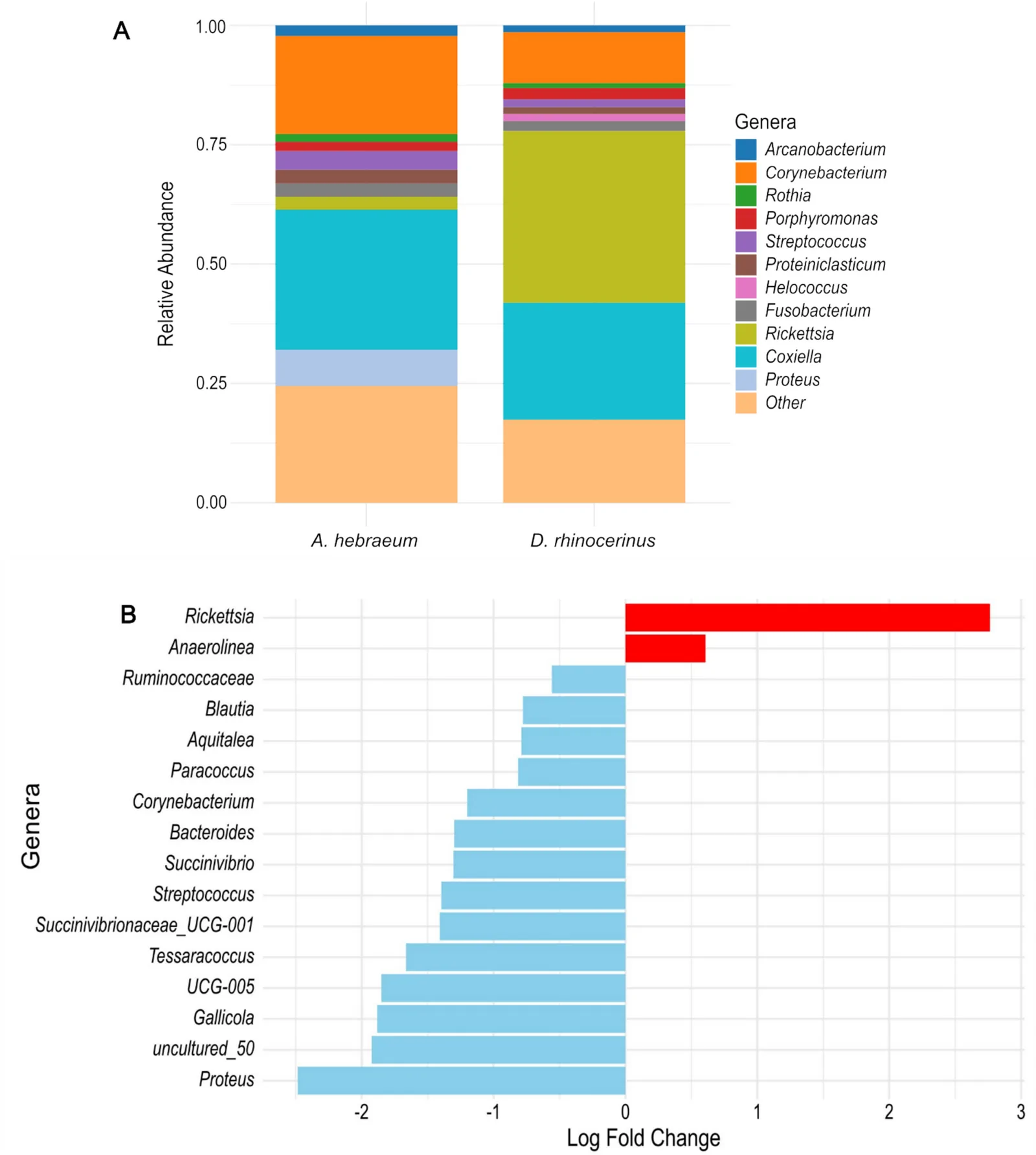
**A** Relative abundance of the 10 most prevalent genera found within *Amblyomma hebraeum* and *Dermacentor rhinocerinus*, respectively. **B** The differential abundance of genera significantly more (red) or less (blue) abundant in *Dermacentor rhinocerinus* in comparison to *Amblyomma hebraeum*

After filtering and correcting for contaminants, a total of 3108 ASVs with a mean sequence read length of 412.33 (± 25.9) bp were characterized, and these belonged to 306 families and 555 genera. All data are available from the NCBI Sequence Read Archive under Bioproject accession number PRJNA1220073. Proteobacteria, Actinobacteria, and Firmicutes were the dominant phyla (Fig. 1). The relative abundance of the 10 most prevalent genera differed between tick species (Fig. 2A). *Rickettsia* (*W* = 4.47, Holm’s adjusted *p*-value = 0.002) and *Anaerolinea* (*W* = 2.75, Holm’s adjusted *p*-value < 0.001) were significantly more abundant in *D. rhinocerinus* than in *A. hebraeum* (Fig. 2B)*.* Furthermore, *Helococcus*, not featuring prominently in *A. hebraeum*, formed part of the 10 most prevalent genera within *D. rhinocerinus. Amblyomma hebraeum* had higher relative abundances of *Corynebacterium*, *Coxiella*, *Proteus*, and *Streptococcus* (Supplementary Fig. 3) with *Proteus*, nearly absent from *Dermacentor.* Differential abundance analysis revealed that *Rickettsia* and *Anaerolinea* were significantly more abundant in *D. rhinocerinus* when compared to *A. hebraeum* (Fig. 2B).

**Fig. 3:**
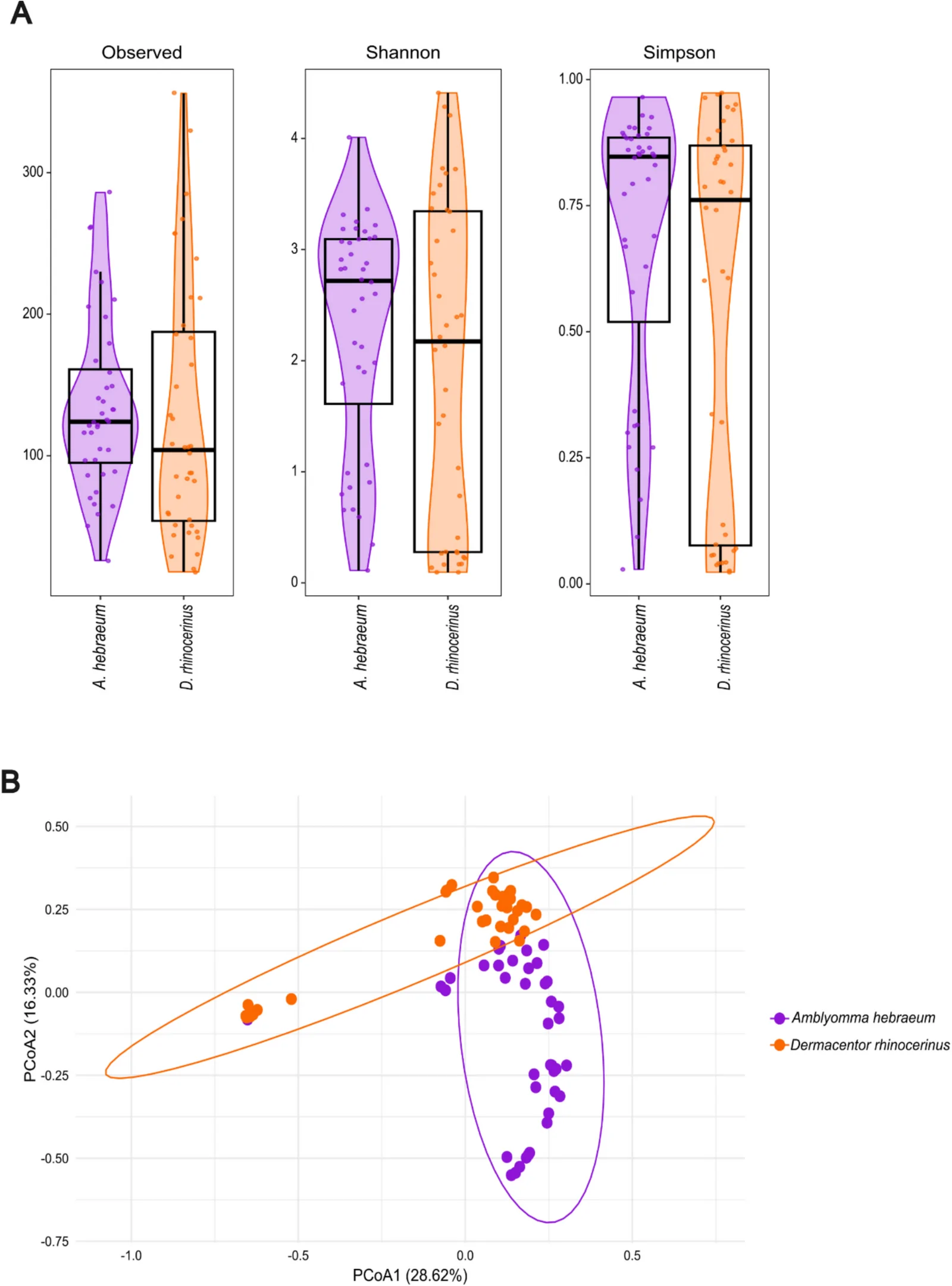
**A** Alpha diversity indices (observed richness, Shannon and Simpson diversity indexes) compared between tick species. For each index, violin plots show the distribution of diversity values, with overlaid boxplots indicating the summary statistics. Individual data points within each violin plot represent sample values. Wilcoxon rank-sum test *p*-values were > 0.05 for all indices. **B** Beta diversity of the microbiome in *Amblyomma hebraeum* (purple) and *Dermacentor rhinocerinus* (orange) displayed by PCoA for distances across samples calculated using Bray–Curtis dissimilarity. Plot ellipses represent 95% confidence regions for the clusters

Although alpha diversity metrics seemed to be generally higher in *A. hebraeum* (Fig. 3A), there were no significant differences between the two tick species for observed richness (*W* = 932, *p* = 0.21), and the Shannon (*W* = 895, *p* = 0.37) and Simpson (*W* = 947, *p* = 0.16) diversity indexes. Beta diversity analysis using Bray–Curtis dissimilarity distances indicated a grouping or clustering of data points according to tick species, suggesting differences in community composition between the two tick species (Fig. 3B) that was significantly supported by PERMANOVA (species: *F* = 6.063, *R*^2^ = 0.073, *p* = 0.001)*.*

### Regional Diversity

Alpha diversity indices showed no significant differences between *A. hebraeum* from the central and from the southern region in the KNP (observed richness: *W* = 0.163.5, *p* = 0.68; Shannon: *W* = 147, *p* = 0.94 and Simpson: *W* = 157, *p* = 0.84). The same result was obtained for *D. rhinocerinus* (observed richness: *W* = 158.5, *p* = 0.80, Shannon: *W* = 157, *p* = 0.84 and Simpson: *W* = 163, *p* = 0.70). The PCoA plot revealed a complete overlap of regional clusters, with the southern cluster falling within the central cluster for both tick species (Supplementary Fig. 4), and PERMANOVA results (region: *F* = 0.3738, *R*^2^ = 0.004, *p* = 0.98) confirmed that regional bacterial community compositions were not significantly different from each other.

### Phylogenetic and Diversity Analyses of *Coxiella* ASVs

**Fig. 4:**
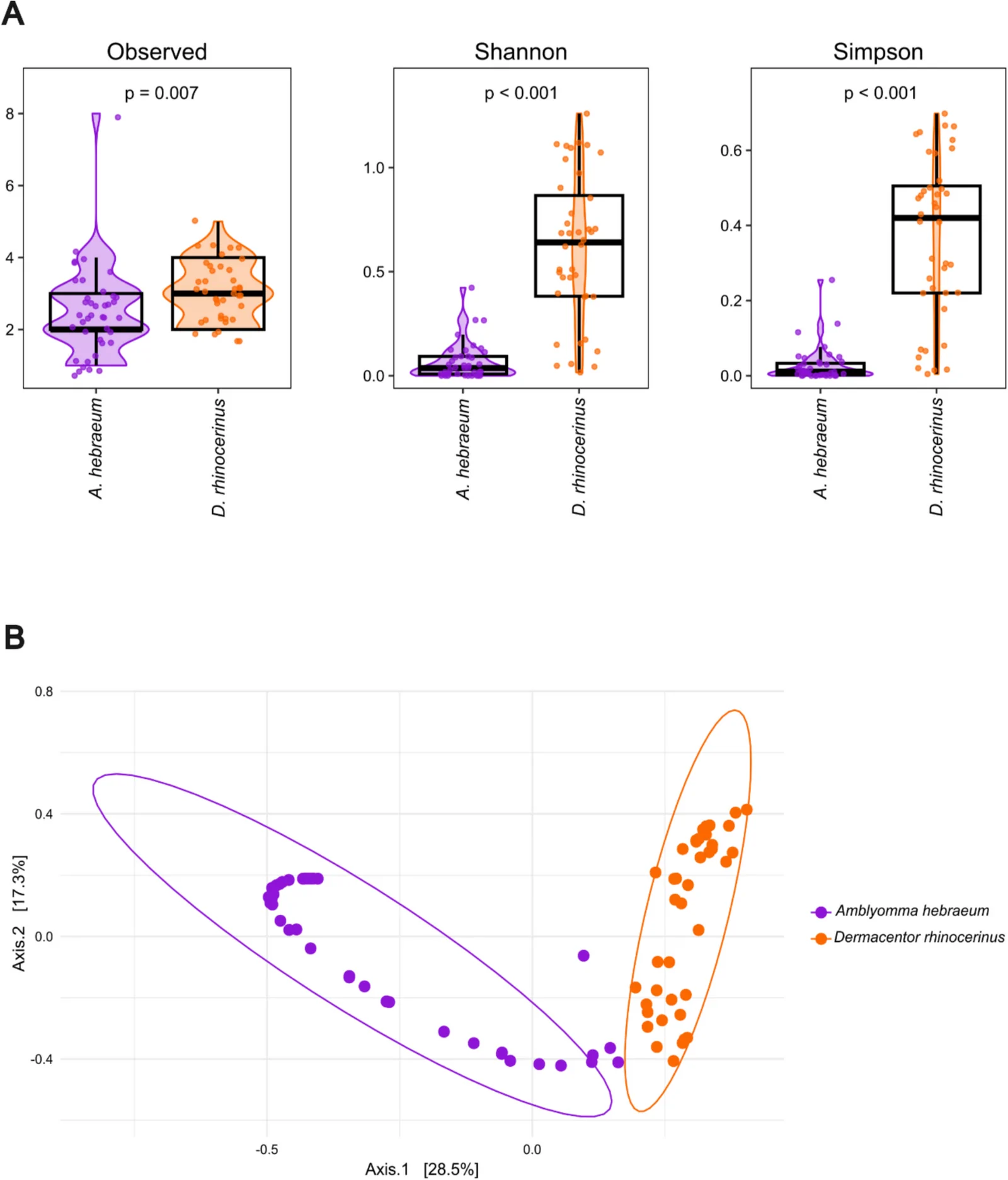
**A** Observed richness, Shannon and Simpson diversity indices for *Coxiella* across tick species. For each index, violin plots show the distribution of diversity values, with overlaid boxplots indicating the summary statistics. Individual data points within each violin plot represent sample values. Significant differences, as indicated by *p*-values from Wilcoxon rank-sum tests, are displayed above the plots. **B** Beta diversity of *Coxiella* found within *Amblyomma hebraeum* (purple) and *Dermacentor rhinocerinus* (orange) displayed by PCoA for distances across samples calculated using Bray–Curtis dissimilarity. Plot ellipses represent 95% confidence regions for the clusters

Twenty-five distinct 16S rRNA *Coxiella* ASV’s were identified. Overall observed richness (*W* = 531.5, *p* = 0.007) and Shannon (*W* = 98, *p* < 0.001) and Simpson (*W* = 94, *p* < 0.001) diversity of these *Coxiella* ASVs were significantly greater in *D. rhinocerinus* compared to *A. hebraeum* (Fig. 4A). The PCoA plot showed clear clustering and separation according to tick species (Fig. 4B) and the PERMANOVA analysis confirmed that the two tick species had significantly different *Coxiella* community compositions (species: *F* = 25.131, *R*^2^ = 0.243, *p* = 0.001).

**Fig. 5:**
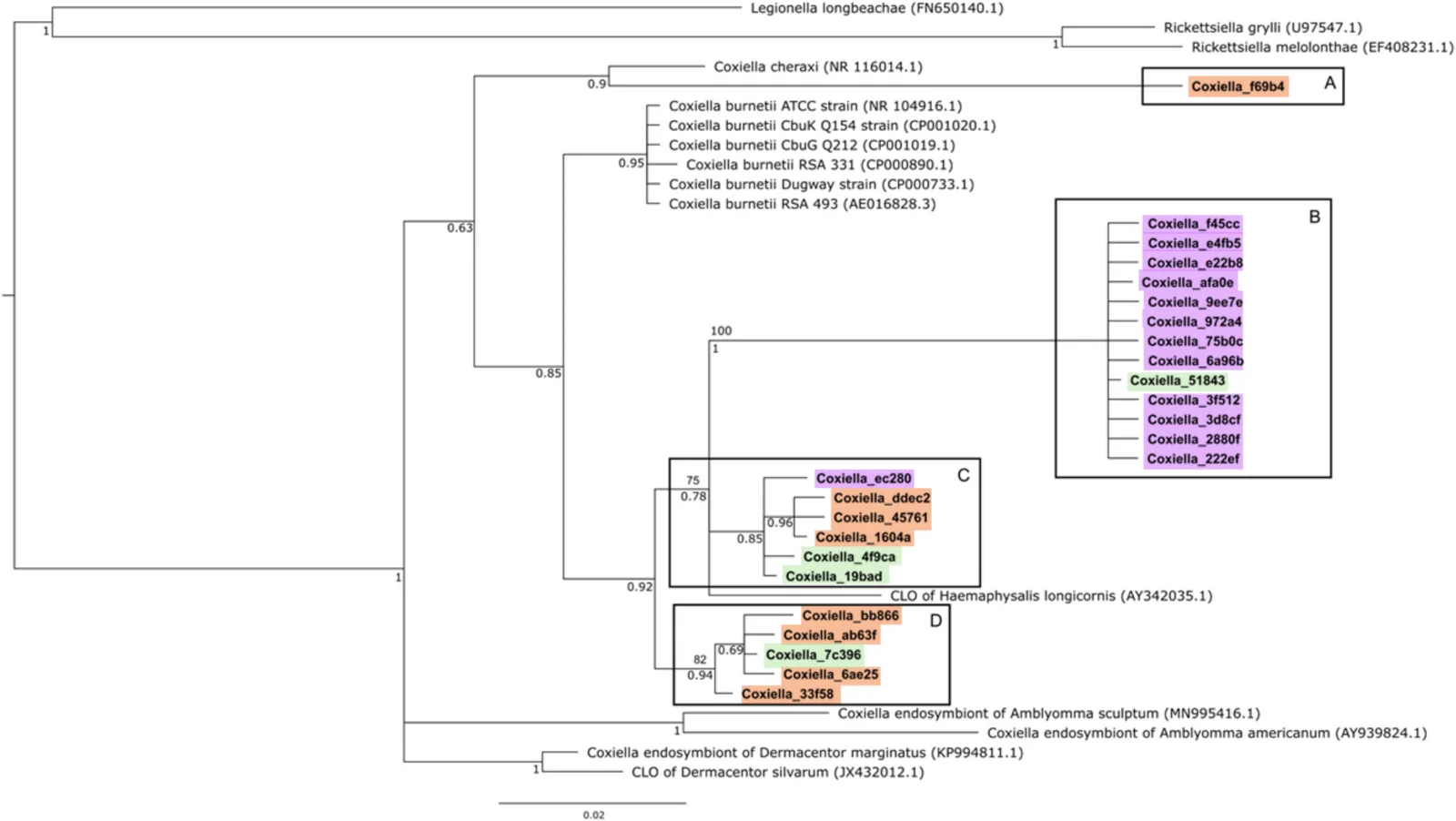
Bayesian inference tree of the phylogenetic relationships of partial 16S rRNA *Coxiella* sequences (433 aligned nucleotide sites). Sequences from the current study are in bold, and the accession numbers of reference sequences are indicated in brackets. Branch numbers indicate Bayesian posterior probabilities (bottom) and the maximum-likelihood (ML) percentage bootstrap support values (top) for each lineage (**A**–**D**) are indicated above the branches. ASVs specific to *Amblyomma hebraeum* are highlighted in purple, those specific to *Dermacentor rhinocerinus* are highlighted in orange, and those shared between both tick species are highlighted in green. *Legionella* and *Rickettsiella* sequences were used as outgroups

The GTR model of sequence evolution was used in the phylogenetic analyses that supported the clustering of all 25 *Coxiella* ASVs in a strongly supported monophyletic group (Fig. 5)*.* Both ML and BI grouped the *Coxiella* ASVs into four distinct lineages (three monophyletic clades and one ASV clustered on its own) with moderate to high support for the nodes defining the four lineages (Fig. 5 and Supplementary Fig. 5). Unfortunately, the phylogenetic analyses could not conclusively resolve which of these four lineages contains the pathogenic *C. burnetii* (due to low bootstrap support values). The individual *Coxiella* ASV, labeled lineage A, was most similar to *C. cheraxi.* The remaining three clades (lineages B–D; Fig. 5) showed some level of association with tick species, with 13 ASVs specific to *A. hebraeum*, eight specific to *D. rhinocerinus*, and four shared by both tick species (Supplementary Fig. 6). The 13 ASVs unique to clade B were nearly all derived from *A. hebraeum*, while clades C and D were mostly derived from *D. rhinocerinus* (Fig. 5). The shared ASVs had higher prevalences relative to the species-specific ASVs in both tick species (Supplementary Fig. 7). Of the shared ASVs, “Coxiella_51843” had the highest prevalence in *A. hebraeum*, found in 100% of the samples, while “Coxiella_7c396” was found in 100% of the samples in *D. rhinocerinus.*

### *Coxiella burnetii* Prevalence

*Coxiella burnetii* was detected in 145/238 individual ticks, 60.9% [95% CI, 54.4–67.1%] (Supplementary Fig. 8). BLASTn of eight sequenced amplicons revealed 99–100% sequence similarity to *C. burnetii. Dermacentor rhinocerinus* showed a higher mean prevalence of positive samples at 66.1% (95% CI, 56.7–74.4%) when compared to *A. hebraeum* with 55.8% (95% CI, 46.5–64.8%), albeit not significantly different (χ^2^ = 2.221, *p* = 0.14) (Supplementary Fig. 9). The overlap in confidence intervals combined with the results from the Fisher’s exact test revealed that there was no significant association between *C. burnetii* prevalence and sampling region (Supplementary Fig. 10).

## Discussion

### Bacterial Community Compositions

This study provided first insights into the rich and diverse bacterial microbiome found within *A. hebraeum* and *D. rhinocerinus* ticks that are associated with white rhinoceros in the KNP, South Africa. Although it has been predicted in the literature that a higher exposure to different hosts during different life stages (such as for *A. hebraeum*) will increase microbiome diversities in ticks [46, 47], this was not supported by our data. The generalist *A. hebraeum* with a larger host range (scrub hares, wild carnivores, small antelope, and game birds) showed a similar species diversity to the host-specific *D. rhinocerinus* (where adults are confined to rhinoceros and nymphs and larvae attach to rodents; [46, 47]). These findings rather support the notion that tick microbiomes are better correlated to species-specific and environmental factors than to host blood source [28]. Although differences in species diversity were not statistically significant, *A. hebraeum* exhibited higher species richness and evenness than *D. rhinocerinus* (Fig. 3A), suggesting a more stable and resilient microbiome. Greater microbial diversity and balance may enhance resistance to disturbance and support functional stability.

In contrast to the lack of significant differences in alpha diversity among the two tick species sampled herein, bacterial community compositions differed significantly between *A. hebraeum* and *D. rhinocerinus.* While *Rickettsia* was the most common bacterium detected in *A. hebraeum* sampled from cattle in a previous study in South Africa [23], *Coxiella* was the dominant genus in *A. hebraeum* collected from rhinoceros hosts in a different geographic region of the country. *Rickettsia* was, however, the most common and significantly more abundant genus within *D. rhinocerinus.* The high incidence of *Coxiella* and *Rickettsia* in *A. hebraeum* and *D. rhinocerinus*, respectively, is not surprising [1, 5, 48] and highlights the importance of these bacteria as endosymbionts for tick survival [3, 33]. While the drivers of the differences in microbial community composition among the two tick species cannot be deduced from our study design, it is tempting to speculate that exposure to different host groups occurring in different environments [47], inter-bacterial competition of the microbes making up the microbiome [49], and the time that various life stages spent feeding on the various hosts [46] could all be invoked as potential mechanisms that play a role in the detected significant differences in beta diversity among *D. rhinocerinus* and *A. hebraeum*.

The present study spans two geographic regions (southern and central regions) which are characterized by differences in precipitation and soil types that also culminate in differences in vegetation patterns and wildlife compositions [50]. A previous study on microbes (Feline Immunodeficiency Virus in lion, *Panthera leo*) showed regional differences among eco-regions [51] and microbiome composition has been reported to differ between geographic regions in other studies [52, 53]. In the present study, there were no significant differences between ticks sampled in the southern and central regions within KNP. At small regional scales, it has been suggested that environmental and host variables play a lesser role in microbiome composition and a few ectoparasite microbiome studies have highlighted the greater influence of species identity on microbiome composition rather than environmental conditions [26, 28, 49]. We propose that the small geographic scale of sampling in our study resulted in the absence of geographical differences in the microbiomes studied herein (also see [54]).

### *Coxiella* Endosymbionts and Pathogens

*Dermacentor rhinocerinus* ticks appeared to have a higher prevalence of pathogenic *C. burnetii* (66.1%) when compared to *A. hebraeum* (55.8%), and interestingly also had a greater diversity of *Coxiella* ASVs than *A. hebraeum* (as evident in branch lengths among ASVs in Fig. 5). The in-depth analyses of the 25 distinct *Coxiella* 16S rRNA ASVs emphasize the high level of sequence diversity among the members of the genus, and the phylogenetic clustering into four distinct lineages/clades that are also associated with specific tick species provides good support for the notion that symbiotic *Coxiella* most likely shared a long convergent evolutionary history with *Amblyomma* and *Dermacentor*, respectively*.* Beta diversity analysis further supported the high level of variation by also displaying significant differences in the community compositions of *Coxiella* ASVs among the tick species. The high prevalence of *Coxiella* within *Amblyomma* and *Dermacentor* may be explained by the *Coxiella* endosymbionts being maternally inherited [13]. Indeed, vertical transmission of *Coxiella* symbionts through the egg has been shown in *Amblyomma cajennense* [55] and Duron et al. [13] also found closely related *Coxiella* endosymbionts present among closely related tick species. However, since the host rhinoceroses were not tested in this study for their microbial community, the transfer of microbial communities between ticks and hosts remains unclear and requires further study.

The greater percentage of species-specific *Coxiella* ASVs detected in *A. hebraeum* compared to *D. rhinocerinus* may be linked to the high functional diversity associated with tick life history*. Coxiella* symbionts would be beneficial for a generalist species—enabling it to feed on multiple host blood sources while obtaining the necessary nourishment. Important to note, however, these unique ASVs had a low prevalence and indicates that they are not widespread among all the individuals sampled herein. While *D. rhinocerinus* had fewer species-specific *Coxiella* ASVs, alpha diversity measures were significantly greater than those of *A. hebraeum*. These results provide circumstantial evidence that *Coxiella* endosymbionts are not passed on from the rhinoceros to the ticks and rather reflect a pattern of host specific co-evolution enforced by vertical transmission within tick species [55].

While it remains untested whether ticks became infected with the pathogenic *C. burnetii* after feeding on an infected rhinoceros host, and/or whether they were carrying the bacterium before attaching to the rhinoceros, variation in host preference during the juvenile life stages of the ticks, and the associated co-evolution between symbiont and host, could provide a potential reason for the higher overall prevalence of *Coxiella* observed in *D. rhinocerinus* [56]. In fact rodents, the preferred hosts for immature *D. rhinocerinus*, are known to play a significant role in *C. burnetii* transmission [21, 57] while there is less known about the role of hosts preferred by immature *A. hebraeum* (e.g., scrub hares, wild carnivores, small antelope, and game birds)*.*

The overall prevalence of *C. burnetii* positive tick samples at 60.9% is slightly lower than the 71% seropositivity previously detected in white rhinoceros sampled in KNP between 2017 and 2019 [16]. This prevalence is higher than that reported for dogs (41.0%; [19]) and domestic ruminants (20.0–68.0%; [19]) in South Africa but to be expected since wild animals often have higher tick burdens than domestic livestock [58]. A higher tick burden could provide more opportunity for *C. burnetii* transmission and persistence within the tick vector populations [16]. Although it is not possible to directly link individual rhinoceros seropositivity to the prevalence detected in the ticks in the current study, results indicate that the potential for *C. burnetii* infection in the KNP white rhinoceros population is high. Circumstantial evidence based on overall *Coxiella* prevalence throughout the park suggests that the pathogenic bacterium may have a similar environmental load throughout the park; however, more research targeting more tick species as well as wildlife host species (and including the northern region of KNP) is required to confirm this observation.

## Supplementary Information

Below is the link to the electronic supplementary material.

Supplementary file1 (DOCX 2300 KB)

## Data Availability

All data are available under Bioproject accession number PRJNA1220073.

## References

[1] Bonnet SI, Pollet T (2021) Update on the intricate tango between tick microbiomes and tick-borne pathogens. Par Immunol 43:e12813. 10.1111/PIM.12813 10.1111/pim.12813 33314216

[2] Qiu Y, Simuunza M, Kajihara M et al (2022) Detection of tick-borne bacterial and protozoan pathogens in ticks from the Zambia-Angola border. Pathogens 11:566. 10.3390/pathogens11050566 35631087 10.3390/pathogens11050566 PMC9144998

[3] Narasimhan S, Fikrig E (2015) Tick microbiome: the force within. Trends Parasitol 31:315–323. 10.1016/j.pt.2015.03.010 25936226 10.1016/j.pt.2015.03.010 PMC4492851

[4] Vayssier-Taussat M, Kazimirova M, Hubalek Z et al (2015) Emerging horizons for tick-borne pathogens: from the ‘one pathogen–one disease’ vision to the pathobiome paradigm. Future Microbiol 10:2033–2043. 10.2217/FMB.15.114 26610021 10.2217/fmb.15.114 PMC4944395

[5] Olivieri E, Kariuki E, Floriano et al (2021) Multi-country investigation of the diversity and associated microorganisms isolated from tick species from domestic animals, wildlife and vegetation in selected African countries. Exp Appl Acarol 83:427–448. 10.1007/S10493-021-00598-3 33646482 10.1007/s10493-021-00598-3 PMC7940270

[6] Porter SR, Czaplicki G, Mainil J et al (2011) Q fever: Current state of knowledge and perspectives of research of a neglected zoonosis. Int J Microbiol 2011:248418. 10.1155/2011/248418 22194752 10.1155/2011/248418 PMC3238387

[7] Eldin C, Mélenotte C, Mediannikov O et al (2017) From Q fever to *Coxiella burnetii* infection: a paradigm change. Clin Microbiol Rev 30:115–190. 10.1128/CMR.00045-16 27856520 10.1128/CMR.00045-16 PMC5217791

[8] Rahravani M, Moravedji M, Mostafavi E et al (2022) The epidemiological survey of *Coxiella burnetii* in small ruminants and their ticks in western Iran. BMC Vet Res 18:292. 10.1186/s12917-022-03396-0 35902914 10.1186/s12917-022-03396-0 PMC9336079

[9] Maurin M, Raoult D (1999) Q Fever. Clin Microbiol Rev 12:518–553. 10.1128/CMR.12.4.518 10515901 10.1128/cmr.12.4.518 PMC88923

[10] Diseko LJ, Tsotetsi-Khambule AM, Onyiche TGE et al (2024) *Coxiella burnetii* infections from animals and ticks in South Africa: a systematic review. Vet Res Comm 48:19–28. 10.1007/s11259-023-10204-z 10.1007/s11259-023-10204-z PMC10811037 37642820

[11] Kamani J, Gonçalves-Oliveira J, Janssen JN et al (2024) Microbiome of two adult tick species and their laboratory-reared offspring shows intra- and inter-species differences. Acta Trop 257:107315. 10.1016/J.ACTATROPICA.2024.107315 38969320 10.1016/j.actatropica.2024.107315

[12] Mangena ML, Gcebe N, Thompson PN, Adesiyun AA (2023) Q fever and toxoplasmosis in South African livestock and wildlife: a retrospective study on seropositivity, sporadic abortion, and stillbirth cases in livestock caused by *Coxiella burnetii*. BMC Vet Res 19. 10.1186/s12917-023-03645-w 10.1186/s12917-023-03645-w PMC10512517 37735412

[13] Duron O, Noël V, McCoy KD et al (2015) The recent evolution of a maternally-inherited endosymbiont of ticks led to the emergence of the Q fever pathogen Coxiella burnetii. PLoS Pathogens 11:e1004892. 10.1371/journal.ppat.1004892 25978383 10.1371/journal.ppat.1004892 PMC4433120

[14] Narasimhan S, Swei A, Abouneameh S et al (2021) Grappling with the tick microbiome. Trends Parasitol 37:722–733. 10.1016/j.pt.2021.04.004 33962878 10.1016/j.pt.2021.04.004 PMC8282638

[15] Emslie R (2020) *Diceros bicornis*. The IUCN Red List of Threatened Species 2020, e.T6557A152728945. 10.2305/IUCN.UK.2020- 1.RLTS.T6557A152728945.en. Accessed 15 Feb 2023.

[16] Donnelly KA, Miller MA, Grobler D et al (2021) Serological evidence of *Coxiella burnetii* infection in the white rhinoceros (*Ceratotherium simum*) in South Africa. J Zoo Wildl Med 52:573–579. 10.1638/2020-0154 34130400 10.1638/2020-0154

[17] Bercier M, LaDouceur EEB, Citino S (2021) Clinical findings, pathology, biosecurity, and serosurveillance of coxiellosis in white rhinoceroses (*Ceratotherium simum*) at a conservation center: two cases. J Zoo Wildl Med 52:389–395. 10.1638/2020-0081 33827203 10.1638/2020-0081

[18] Mtshali K, Khumalo ZTH, Nakao R et al (2015) Molecular detection of zoonotic tick-borne pathogens from ticks collected from ruminants in four South African provinces. J Vet Med Sci 77:1573–1579. 10.1292/jvms.15-0170 26227797 10.1292/jvms.15-0170 PMC4710712

[19] Mtshali K, Nakao R, Sugimoto C, Thekisoe O (2017) Occurrence of *Coxiella burnetii*, *Ehrlichia canis*, *Rickettsia* species and *Anaplasma phagocytophilum*-like bacterium in ticks collected from dogs and cats in South Africa. J SA Vet Ass 88:e1–e6. 10.4102/jsava.v88i0.1390 10.4102/jsava.v88i0.1390 PMC6138182 28582983

[20] Guo H, Adjou Moumouni PF, Thekisoe O et al (2019) Genetic characterization of tick-borne pathogens in ticks infesting cattle and sheep from three South African provinces. Ticks Tick-Borne Dis 10:875–882. 10.1016/j.ttbdis.2019.04.008 31010732 10.1016/j.ttbdis.2019.04.008

[21] Horak IG, Williams R, Heyne H et al (2018) The ixodid ticks (Acari: Ixodidae) of Southern Africa, 1st edn. Springer

[22] Horak IG, Fourie LJ, Heyne H et al (2002) Ixodid ticks feeding on humans in South Africa: with notes on preferred hosts, geographic distribution, seasonal occurrence and transmission of pathogens. Exp Appl Acarol 27:113–136. 10.1023/A:1021587001198/METRICS 12593517 10.1023/a:1021587001198

[23] Kisten D, Brinkerhoff J, Tshilwane SI, Mukaratirwa S (2021) A pilot study on the microbiome of *Amblyomma hebraeum* tick stages infected and non-infected with *Rickettsia africae*. Pathogens 10:941. 10.3390/pathogens10080941 34451405 10.3390/pathogens10080941 PMC8398150

[24] Mazhetese E, Lukanji Z, Byaruhanga C et al (2022) *Rickettsia africae* infection rates and transovarial transmission in *Amblyomma hebraeum* ticks in Mnisi, Bushbuckridge, South Africa. Exp Appl Acarol 86:407–418. 10.1007/S10493-022-00696-W/FIGURES/2 35212871 10.1007/s10493-022-00696-w

[25] Földvári G, Široký P, Szekeres S et al (2016) *Dermacentor reticulatus*: a vector on the rise. Parasites Vectors 9:314. 10.1186/s13071-016-1599-x 27251148 10.1186/s13071-016-1599-x PMC4888597

[26] Hawlena H, Rynkiewicz E, Toh E et al (2013) The arthropod, but not the vertebrate host or its environment, dictates bacterial community composition of fleas and ticks. ISME J 7:221–223. 10.1038/ismej.2012.71 22739493 10.1038/ismej.2012.71 PMC3526175

[27] Chicana B, Couper LI, Kwan JY, Tahiraj E, Swei A (2019) Comparative microbiome profiles of sympatric tick species from the far-western United States. Insects 10:353. 10.3390/insects10100353 31635285 10.3390/insects10100353 PMC6836157

[28] Kueneman JG, Esser HJ, Weiss SJ et al (2021) Tick microbiomes in neotropical forest fragments are best explained by tick-associated and environmental factors rather than host blood source. Appl Environ Microbiol 87:e02668-e2720. 10.1128/AEM.00777-21 33514519 10.1128/AEM.02668-20 PMC8091620

[29] van Treuren W, Ponnusamy L, Brinkerhoff RJ et al (2015) Variation in the microbiota of Ixodes ticks with regard to geography, species, and sex. Appl Environ Microbiol 81:6200–6209. 10.1128/AEM.01562-15 26150449 10.1128/AEM.01562-15 PMC4542252

[30] René-Martellet M, Minard G, Massot R et al (2017) Bacterial microbiota associated with Rhipicephalus sanguineus (s.l.) ticks from France Senegal and Arizona. Parasites Vectors 10:416. 10.1186/s13071-017-2352-9 28886749 10.1186/s13071-017-2352-9 PMC5591579

[31] Couret J, Schofield S, Narasimhan S (2022) The environment, the tick, and the pathogen – it is an ensemble. Front Cell Infec Microbiol 12:1049646. 10.3389/FCIMB.2022.1049646/BIBTEX 36405964 10.3389/fcimb.2022.1049646 PMC9666722

[32] Li SS, Zhang XY, Zhou XJ et al (2022) Bacterial microbiota analysis demonstrates that ticks can acquire bacteria from habitat and host blood meal. Exp Appl Acarol 87:81–95. 10.1007/s10493-022-00714-x 35532740 10.1007/s10493-022-00714-x

[33] Bonnet SI, Binetruy F, Hernández-Jarguín AM, Duron O (2017) The tick microbiome: why non-pathogenic microorganisms matter in tick biology and pathogen transmission. Front Cell Infec Microbiol 7:236. 10.3389/fcimb.2017.00236 28642842 10.3389/fcimb.2017.00236 PMC5462901

[34] Arbeitskreis Blut U, Bewertung BK (2014) *Coxiella burnetii* - pathogenic agent of Q (query) fever. Transf Med Hemother 41:60–72. 10.1159/000357107 10.1159/000357107 PMC3949614 24659949

[35] Mori H, Maruyama F, Kato H et al (2013) Design and experimental application of a novel non-degenerate universal primer set that amplifies prokaryotic 16S rRNA genes with a low possibility to amplify eukaryotic rRNA genes. DNA Res 21:217–227. 10.1093/dnares/dst052 24277737 10.1093/dnares/dst052 PMC3989492

[36] Kamau MW, Witte C, Goosen W et al (2024) Comparison of 72 test performance of a conventional PCR and two field-friendly tests to detect *Coxiella burnetii* DNA in ticks using Bayesian latent class analysis. Front Vet Sci 11:1396714. 10.3389/FVETS.2024.1396714/BIBTEX 38962707 10.3389/fvets.2024.1396714 PMC11220323

[37] Andrews S (2010) FastQC: a quality control tool for high throughput sequence data [Online]. Available online at: http://www.bioinformatics.babraham.ac.uk/projects/fastqc/

[38] Ewels P, Magnusson M, Lundin S, Käller M (2016) MultiQC: summarize analysis results for multiple tools and samples in a single report. Bioinformatics 32:3047–3048. 10.1093/bioinformatics/btw354 27312411 10.1093/bioinformatics/btw354 PMC5039924

[39] Bolyen E, Rideout JR, Dillon MR et al (2019) Reproducible, interactive, scalable and extensible microbiome data science using QIIME 2. Nature Biotech 37:852–857. 10.1038/s41587-019-0209-9 10.1038/s41587-019-0209-9 PMC7015180 31341288

[40] McMurdie PJ, Holmes S (2013) Phyloseq: an R package for reproducible interactive analysis and graphics of microbiome census data. PLoS ONE 8. 10.1371/journal.pone.0061217. 10.1371/journal.pone.0061217 PMC3632530 23630581

[41] Wickham H (2016). *ggplot2: Elegant graphics for data analysis.* Springer-Verlag New York. ISBN 978-3-319-24277-4

[42] Oksanen J, Simpson G, Blanchet F et al (2024) _vegan: Community Ecology Package_. R package version 2.6–6.1, <https://CRAN.R-project.org/package=vegan>

[43] Lin H, Eggesbø M, Das PS (2022) Linear and nonlinear correlation estimators unveil undescribed taxa interactions in microbiome data. Nature Comm 13:4964. 10.1038/s41467-022-32243-x 10.1038/s41467-022-32243-x PMC9399263 35999204

[44] Tamura K, Stecher G, Kumar S (2021) MEGA11: Molecular Evolutionary Genetics Analysis version 11. Mol Biol Evol 38:3022–3027. 10.1093/molbev/msab120 33892491 10.1093/molbev/msab120 PMC8233496

[45] Ronquist F, Teslenko M, Van Der Mark P et al (2012) MrBayes 3.2: efficient Bayesian phylogenetic inference and model choice across a large model space. Syst Biol 61:539–542. 10.1093/sysbio/sys029 22357727 10.1093/sysbio/sys029 PMC3329765

[46] Menchaca AC, Visi DK, Strey OF et al (2013) Preliminary assessment of microbiome changes following blood-feeding and survivorship in the *Amblyomma americanum* nymph-to-adult transition using semiconductor sequencing. PLoS ONE 8:1–10. 10.1371/journal 10.1371/journal.pone.0067129 PMC3691118 23826210

[47] Swei A, Kwan JY (2017) Tick microbiome and pathogen acquisition altered by host blood meal. ISME J 11:813–816. 10.1038/ISMEJ.2016.152 27858931 10.1038/ismej.2016.152 PMC5322304

[48] Hunter DJ, Torkelson JL, Bodnar J et al (2015) The *Rickettsia* endosymbiont of *Ixodes pacificus* contains all the genes of de novo folate biosynthesis. PLoS ONE 10:e0144552. 10.1371/JOURNAL.PONE.0144552 26650541 10.1371/journal.pone.0144552 PMC4674097

[49] Fountain-Jones NM, Khoo BS, Rau A et al (2023) Positive associations matter: microbial relationships drive tick microbiome composition. Mol Ecol 32:4078–4092. 10.1111/MEC.16985 37173817 10.1111/mec.16985

[50] Gertenbach WPD (1983) Landscapes of the Kruger National Park. Koedoe 26:9–121

[51] Kerr TJ, Matthee S, Govender D et al (2018) Viruses as indicators of contemporary host dispersal and phylogeography: an example of feline immunodeficiency virus (FIVPle) in free-ranging African lion (*Panthera leo*). J Evol Biol 31:1529–1543. 10.1111/jeb.13348 29964350 10.1111/jeb.13348

[52] Medeiros MCI, Seabourn PS, Rollins RL, Yoneishi NM (2022) Mosquito microbiome diversity varies along a landscape-scale moisture gradient. Microb Ecol 84:893–900. 10.1007/s00248-021-01865-x 34617123 10.1007/s00248-021-01865-x PMC11233147

[53] Matthee C, Matthee S, Bierman A et al (2023) Documenting the microbiome diversity and distribution in selected fleas from South Africa with an emphasis on the cat flea. Ctenocephalides f felis Parasitology 150:979–989. 10.1017/S0031182023000835 37681253 10.1017/S0031182023000835 PMC10941216

[54] Brinkerhoff RJ, Clark C, Ocasio K et al (2020) Factors affecting the microbiome of *Ixodes scapularis* and *Amblyomma americanum*. PLoS ONE 15:e0232398. 10.1371/JOURNAL.PONE.0232398 32413031 10.1371/journal.pone.0232398 PMC7228056

[55] Machado-Ferreira E, Dietrich G, Hojgaard A et al (2011) *Coxiella* symbionts in the cayenne tick *Amblyomma cajennense*. Micro Ecol 62:134–142. 10.1007/s00248-011-9868-x 10.1007/s00248-011-9868-x 21611689

[56] González J, González MG, Valcárcel F et al (2020) Prevalence of *Coxiella burnetii* (Legionellales: *Coxiellaceae*) infection among wildlife species and the tick *Hyalomma lusitanicum* (Acari: *Ixodidae*) in a meso Mediterranean ecosystem. J Med Entomol 57:551–556. 10.1093/jme/tjz169 31589748 10.1093/jme/tjz169

[57] Abdel-Moein KA, Hamza DA (2018) Rat as an overlooked reservoir for *Coxiella burnetii*: a public health implication. Comp Immuno, Microbiol Infect Dis 61:30–33. 10.1016/j.cimid.2018.11.002 10.1016/j.cimid.2018.11.002 30502830

[58] Ndeereh D, Muchemi G, Thaiyah A et al (2017) Molecular survey of *Coxiella burnetii* in wildlife and ticks at wildlife–livestock interfaces in Kenya. Exp Appl Acarol 72:277–289. 10.1007/s10493017-0146-6 28593481 10.1007/s10493-017-0146-6

