# Supplementary material for: The Microbiome and Coxiella Diversity Found in Amblyomma hebraeum and Dermacentor rhinocerinus Ticks Sampled from White Rhinoceros
Source: Microb Ecol. 2025 May 22;88:48. doi: 10.1007/s00248-025-02549-6 (PMC12098525; doi:10.1007/s00248-025-02549-6)
Supplement: Supplementary file 1 [file 248_2025_2549_MOESM1_ESM.docx]

Table 1: List and accession numbers of the bacterial strains used as reference sequences in the phylogenetic analysis.

| **Bacteria** | **GenBank accession number** | **Reference** |
| --- | --- | --- |
| *Coxiella burnetii* (strain CbuG / Q212) | CP001019 | Beare *et al.* (2009) |
| *Coxiella burnetii* (strain Henzerling / RSA 331) | CP000890 | Unpublished |
| *Coxiella burnetii* (strain CbuK / Q154) | CP001020 | Beare *et al.* (2009) |
| *Coxiella burnetii* (strain Nine Mile I / RSA 493) | AE016828 | Seshadri *et al.* (2003) |
| *Coxiella burnetii* (strain Dugway / 5J108 111) | CP000733 | Beare *et al.* (2009) |
| *Coxiella* endosymbiont of Amblyomma americanum | AY939824 | Unpublished |
| *Coxiella* endosymbiont of *Amblyomma sculptum* isolate CoxAsculpt | MN995416.1 | Binetruy *et al.* (2020) |
| *Coxiella* endosymbiont of *Dermacentor marginatus* isolate Dmar1 | KP994811.1 | Duron *et al.* (2015) |
| *Haemaphysalis longicornis* symbiont 47 | AY342035 | Lee *et al.* (2004) |
| *Coxiella burnetii* strain ATCC VR-615 | NR_104916.1 | Unpublished |
| *Coxiella cheraxi* strain TO-98 | NR_116014.1 | Unpublished |
| CLO of *Dermacentor silvarum* clone Dx-56 | JX432012 | Liu *et al.* (2013) |
| *Coxiella* symbiont of *Carios capensis* clone Scc4 | DQ100452 | Unpublished |
| *Legionella longbeachae* (strain NSW150) | FN650140 | Cazalet *et al.* (2010) |
| *Rickettsiella melolonthae* (strain BBA1806/LAM6-D/2004) | EF408231 | Leclerque and Kleespies (2008) |
| *Rickettsiella grylli* | U97547 | Roux *et al.* (1997) |
| *Rickettsia amblyommii* | U11012 | Unpublished |

Supplementary Figure 1: Map indicating the regions of the Kruger National Park. The number of white rhinoceros individuals sampled in the central and southern regions for the study is noted in parenthesis. The rhinoceros individuals sampled are represented by the black points.

Supplementary Figure 2: Rarefaction curves indicating the effect of sampling depth (read counts per sample) on ASV richness in tick samples.

Supplementary Figure 3: A comparison of the relative abundance of the top 5 genera found within Amblyomma hebraeum and Dermacentor rhinocerinus.

Supplementary Figure 4: Principal coordinate analysis plot for *Amblyomma hebraeum* sampled in the Southern (red circles) and Central (black circles) regions, as well as *Dermacentor rhinocerinus* sampled in the Southern (blue squares) and Central (green squares) regions; distances across samples were calculated using Bray-Curtis distances. Plot ellipses represent 95% confidence regions for the clusters.

Supplementary Figure 5: *Coxiella* phylogeny constructed using Maximum-likelihood (ML) estimations based on partial 16S rRNA gene sequences (433 aligned nucleotide sites). Sequences from the study are in bold and the accession numbers of reference sequences are indicated in brackets. Branch numbers indicate bootstrap support (100 replicates; only bootstrap values >60% are shown). *Legionella* and *Rickettsiella* sequences were used as outgroups.

Supplementary Figure 6: Venn diagram representing the number of shared *Coxiella* ASVs among *Amblyomma hebraeum* and *Dermacentor rhinocerinus.*

Supplementary Figure 7: The prevalence of *Coxiella* ASVs found across *Amblyomma hebraeum* and *Dermacentor rhinocerinus*.

Supplementary Figure 8: Overall prevalence of *Coxiella burnetii* in tick DNA samples screened by *IS1111* transposase element PCR in this study; positive results (red) and negative (blue).

Supplementary Figure 9: Boxplot comparing the prevalence of *Coxiella burnetii* DNA-positive samples between *Amblyomma hebraeum* (purple) and *Dermacentor rhinocerinus* (orange) ticks. Error bars represent the 95% confidence intervals for each species. A Chi-squared test revealed no significant difference in prevalence between the two species (p > 0.05).

Supplementary Figure 10: Prevalence of *Coxiella burnetii* DNA in *Amblyomma hebraeum* and *Dermacentor rhinocerinus* ticks by region of Kruger National Park. Prevalence of ticks sampled in the central region is represented by the pink bars while that from the southern region is represented by the green bars. Error bars represent the 95% confidence intervals. Fisher’s exact test revealed no significant association between region and prevalence for either tick species (p > 0.05).
